# Supplementary material for: Patterns of Intron Gain and Loss in Fungi
Source: PLoS Biol. 2004 Nov 30;2(12):e422. doi: 10.1371/journal.pbio.0020422 (PMC532390; doi:10.1371/journal.pbio.0020422)
Supplement: Table S1 — Also available at http://genes.mit.edu/NielsenEtAl/. (4.3 MB ZIP). [file pbio.0020422.st001.zip › NielsenEtAl/html/1271.html]

AN4280.1.NCU03556.1.MG03247.1.FG00308.1


```
 CLUSTAL W (1.82) Multiple Sequence Alignments - Introns Inserted


Sequence 1: MG03247.1	333 aa
Sequence 2: FG00308.1	345 aa
Sequence 3: NCU03556.1	340 aa
Sequence 4: AN4280.1	320 aa
Alignment Length: 387 aa
Number Identitical Residues: 178 aa
Alignment Score (without introns) 7816


MG03247.1 	-----------------------------MVAASKPVIPQNDNVAHALAGAGGGLLSMIL
NCU03556.1	------------------------------MSQSKPVVPQNDNVAHALAGAGGGILSMAL
FG00308.1 	MSDNVDKETTPEIDSPITKVEITPTKIEIEKAPEVPAIPQNDNVAHALAGAGGGLLSMIL
AN4280.1  	--------------MSLSKVEDP------KKVAAAAAAQQSDNIAHALAGAGGGILSMVL
          	               . :. . .            ..  *.**:**********:*** *

MG03247.1 	T2YPLITISTRAQVEAKQADSKKKAGAFLDAIRTIVAREGASGLYAGLSSALFGITVTNF
NCU03556.1	T2YPLITLSTRAQVESKRSADT----TFLAAVQKIVAREGISGLYSGLSSALFGISVTNF
FG00308.1 	T2YPLITLSTRAQVESKKAESK-----FSEAVGNIIAREGISGLYSGINSALFGISITNF
AN4280.1  	T2YPLITLSTRAQVESKRAQSS-----TIDAIRRIVQREGIVGLYSGLESALFGISVTNF
          	* *****:*******:*:: ..        *:  *: ***  ***:*:.******::***

MG03247.1 	VYYYWYEWTRAFFEKAAVRAGRASSKLTTVESMLAGALAGSATVLITNPIWVVNTRMTTR
NCU03556.1	VYYYWYEWTRAFFEAAAVKAGRASKKLTTVESMIAGAIAGSATVILTNPIWVVNTRMTTR
FG00308.1 	VYYYWYEWTRGFFEKAAAKAGRAGSKLTTVESMIAGAIAGSATVIITNPIWVVNTRVTTR
AN4280.1  	VYYYWYEWTRSAFEKAAEKAGRS-KKLSTLESMIAGAIAGSATVLLTNPIWVVNTRVTAR
          	**********. ** ** :***: .**:*:***:***:******::**********:*:*

MG03247.1 	KQQVARASDDEKAAEEGLADGADKAETSVVKGPKVEPAPGTMATLLALLRHEGPQALFRG
NCU03556.1	KAAAAD-DDDEKNTASALPG------------APPAKKPSTIGTLLALLKNEGPQALFAG
FG00308.1 	QQ------EKKKDVEAGESS-------------QPAKAPSTIGTLLLLLKNEGPQALFAG
AN4280.1  	KS---------AEDDQSLPG------------APKKQRPSTFGTLMDLLQKEGPTALFAG
          	:               . ..            .     *.*:.**: **::*** *** *

MG03247.1 	VMPALVLVINPILQYTLFEQLKNTVEKRRRVTPTVAFFLGALGKLFATSITYPYITVKSQ
NCU03556.1	VVPALVLVINPILQYTLFEQMKNAVEKRRKMTATLAFFLGAAGKLFATSVTYPYITVKSQ
FG00308.1 	VIPALVLVINPILQYTLFEQLKNTVEKRRKVTPAVAFFLGALGKLFATAITYPYITVKSQ
AN4280.1  	VLPALVLVINPILQYTIFEQLKNIVERRRRMTPKDAFYLGALGKILATSITYPYITVKSR
          	*:**************:***:** **:**::*.  **:*** **::**::*********:

MG03247.1 	MHVAD------NGRKEGMTEAMRRVIKEEGYS----G1IGPKVSQSVLTAAFLFAFKDVL
NCU03556.1	MHVAPANDGTGAQKREGMMEAINRVVREEGYAGLYKG1IGPKVTQSVLTAAFLFAFKDVL
FG00308.1 	MHVQ-------SGQKEGSLAALSRIVRESGYSGLYRG1IGPKITQSVLTAALLFAFKDVL
AN4280.1  	MHVASK-----DGPKESLNGSLKRIIKEEGYTGLYKG1IGPKVTQSAITAAFLFAFKDVL
          	***           :*.   :: *:::*.**:.   * ****::**.:***:********

MG03247.1 	YEQTVKLRGT~VARK---------------
NCU03556.1	YEQTVKLRGR0QQQQQQQQQQQQQQQINNN
FG00308.1 	YEQTVRLRMA~QAARRRVA-----------
AN4280.1  	YDAMASLRRS~RVSK---------------
          	*:  . **      :
```
